# Supplementary material for: Potentially inappropriate medication uses and associated factors among elderly primary health care clinics attendees: A call to action
Source: PLoS One. 2023 Aug 24;18(8):e0290625. doi: 10.1371/journal.pone.0290625 (PMC10449131; doi:10.1371/journal.pone.0290625)
Supplement: S1 Checklist — (DOCX) [file pone.0290625.s001.docx]

STROBE Statement—checklist of items that should be included in reports of observational studies

|  | Item No. | Recommendation | Page  No. | Relevant text from manuscript |
| --- | --- | --- | --- | --- |
| **Title and abstract** | 1 | (*a*) Indicate the study’s design with a commonly used term in the title or the abstract | 1 | a descriptive cross-sectional study was conducted………. |
|  |  | (*b*) Provide in the abstract an informative and balanced summary of what was done and what was found | 1-2 | The study included 421 elderly people (197 men and 224 women) with an average age of 73.6 years ……………………….. |
| Introduction | | | |  |
| Background/rationale | 2 | Explain the scientific background and rationale for the investigation being reported | 3 | PIM is a subject of global concern, especially among the elderly, as it is a significant …….  In Palestine, the elderly account for around 3.3% ……………….. |
| Objectives | 3 | State specific objectives, including any prespecified hypotheses | 3 | The primary objective of this study is to measure ………… |
| Methods | | | |  |
| Study design | 4 | Present key elements of study design early in the paper | 3 | An observational descriptive cross-sectional study was ………… |
| Setting | 5 | Describe the setting, locations, and relevant dates, including periods of recruitment, exposure, follow-up, and data collection | 3-4 | among elderly people attending primary health care (PHC) clinic ………… |
| Participants | 6 | (*a*) *Cohort study*—Give the eligibility criteria, and the sources and methods of selection of participants. Describe methods of follow-up  *Case-control study*—Give the eligibility criteria, and the sources and methods of case ascertainment and control selection. Give the rationale for the choice of cases and controls  *Cross-sectional study*—Give the eligibility criteria, and the sources and methods of selection of participants | 4 | The study included all patients ≥ 65 years who had …………… |
|  |  | (*b*) *Cohort study*—For matched studies, give matching criteria and number of exposed and unexposed  *Case-control study*—For matched studies, give matching criteria and the number of controls per case |  |  |
| Variables | 7 | Clearly define all outcomes, exposures, predictors, potential confounders, and effect modifiers. Give diagnostic criteria, if applicable | 4 | It consists of three sections; the first one is about demographic data such as age, gender, weight, residency, ………………… |
| Data sources/ measurement | 8* | For each variable of interest, give sources of data and details of methods of assessment (measurement). Describe comparability of assessment methods if there is more than one group | 4 | We used an interviewer-administered questionnaire for data collection. It …….. |
| Bias | 9 | Describe any efforts to address potential sources of bias | 4 | while most of the data were available in patients' files, still interview was useful to gather more information like medication ….. |
| Study size | 10 | Explain how the study size was arrived at | 4 | A minimum sample size of 387 was calculated ……. |

Continued on next page

| Quantitative variables | 11 | Explain how quantitative variables were handled in the analyses. If applicable, describe which groupings were chosen and why | 5 | Descriptive statistics such as frequency and percentages and their 95% confidence intervals are used to describe patient characteristics, polypharmacy prevalence, and PIM ………….. |
| --- | --- | --- | --- | --- |
| Statistical methods | 12 | (*a*) Describe all statistical methods, including those used to control for confounding | 5 | we used the logistic regression model to assess the variables independently associated with PIM ………….. |
|  |  | (*b*) Describe any methods used to examine subgroups and interactions | 5 | We used the chi-square test to examine the relationship ……… |
|  |  | (*c*) Explain how missing data were addressed | 5 | This study had no missing data due to the use …………. |
|  |  | (*d*) *Cohort study*—If applicable, explain how loss to follow-up was addressed  *Case-control study*—If applicable, explain how matching of cases and controls was addressed  *Cross-sectional study*—If applicable, describe analytical methods taking account of sampling strategy |  |  |
|  |  | (*e*) Describe any sensitivity analyses |  |  |
| Results | | | | |
| Participants | 13* | (a) Report numbers of individuals at each stage of study—eg numbers potentially eligible, examined for eligibility, confirmed eligible, included in the study, completing follow-up, and analysed | 5-9 | A total of 421 geriatric patients of PHC clinics attendants participated in this study………. |
|  |  | (b) Give reasons for non-participation at each stage | N/A |  |
|  |  | (c) Consider use of a flow diagram |  |  |
| Descriptive data | 14* | (a) Give characteristics of study participants (eg demographic, clinical, social) and information on exposures and potential confounders | 5 | Almost half of them (46.8%) were male, 66.3% were married, and 59.1% ……….  Table 1 …………………….. |
|  |  | (b) Indicate number of participants with missing data for each variable of interest |  |  |
|  |  | (c) *Cohort study*—Summarise follow-up time (eg, average and total amount) |  |  |
| Outcome data | 15* | *Cohort study*—Report numbers of outcome events or summary measures over time |  |  |
|  |  | *Case-control study—*Report numbers in each exposure category, or summary measures of exposure |  |  |
|  |  | *Cross-sectional study—*Report numbers of outcome events or summary measures | 7 | The average number of total medications used per patient was six, ranging from zero to 15. About two-thirds of them (66.7%) use 5-9 medications ……….. |
| Main results | 16 | (*a*) Give unadjusted estimates and, if applicable, confounder-adjusted estimates and their precision (eg, 95% confidence interval). Make clear which confounders were adjusted for and why they were included | 8 | Multivariate analysis was used to identify factors independently associated with PIM…….  Table 4 ……………………… |
|  |  | (*b*) Report category boundaries when continuous variables were categorized | 6- 9 | Table 1 and Table 4 |
|  |  | (*c*) If relevant, consider translating estimates of relative risk into absolute risk for a meaningful time period |  |  |

Continued on next page

| Other analyses | 17 | Report other analyses done—eg analyses of subgroups and interactions, and sensitivity analyses |  |  |
| --- | --- | --- | --- | --- |
| Discussion | | | | |
| Key results | 18 | Summarise key results with reference to study objectives | 10 | More than one-third of elderly patients attending PHC clinics in our study were found to use at least one PIM.……… |
| Limitations | 19 | Discuss limitations of the study, taking into account sources of potential bias or imprecision. Discuss both direction and magnitude of any potential bias | 12 | However, some limitations should be taken into consideration……… |
| Interpretation | 20 | Give a cautious overall interpretation of results considering objectives, limitations, multiplicity of analyses, results from similar studies, and other relevant evidence | 9-12 | Taking into consideration the fact that 56.3 % of study participants…. |
| Generalisability | 21 | Discuss the generalisability (external validity) of the study results | 12 | We believe that the findings of this study are generalizable to the ….. |
| Other information | |  | | |
| Funding | 22 | Give the source of funding and the role of the funders for the present study and, if applicable, for the original study on which the present article is based | -- | According to the journal Submission Guidelines, funding information is included in the online submission information. |

*Give information separately for cases and controls in case-control studies and, if applicable, for exposed and unexposed groups in cohort and cross-sectional studies.

**Note:** An Explanation and Elaboration article discusses each checklist item and gives methodological background and published examples of transparent reporting. The STROBE checklist is best used in conjunction with this article (freely available on the Web sites of PLoS Medicine at http://www.plosmedicine.org/, Annals of Internal Medicine at http://www.annals.org/, and Epidemiology at http://www.epidem.com/). Information on the STROBE Initiative is available at www.strobe-statement.org.
