# Supplementary material for: Potentially inappropriate medication uses and associated factors among elderly primary health care clinics attendees: A call to action
Source: PLoS One. 2023 Aug 24;18(8):e0290625. doi: 10.1371/journal.pone.0290625 (PMC10449131; doi:10.1371/journal.pone.0290625)
Supplement: Attachment — Submitted filename: Response to Reviewers.docx [file pone.0290625.s003.docx]

DATE: 03\08\2023

**Dear Dr. Muhammad Eid Akkawi**

**Academic Editor/ PLOS ONE**

RE: Manuscript entitled "Potentially inappropriate medication uses and associated factors among elderly primary health care clinics attendees: a call to action" ([PONE-D-23-10547] - [EMID:c0429b4b8a8af4b0]).

We appreciate your time and effort in evaluating the reviewers' comments and responding to us.

We appreciate yours and the reviewers' comments. We carefully read each reviewer's comments and the

Journal Requirements and responded to them point by point below.

We submitted one clean copy and a marked-up copy of our manuscript that highlights changes made to the original version. Our responses below are in the “Authors’ Response” column and shown in blue to distinguish them from the Reviewers’ comments.

We hope that we have adequately responded to the reviewers' concerns. We believe that this has strengthened our paper and look forward to hearing from you. Please let us know if you have any additional suggestions or if anything is unclear.

Many thanks for considering our revision for publication.

Sincerely,

Zaher Nazzal

| **Reviewer 1 comments:** | **Authors’ Response:** |
| --- | --- |
| It is an interesting work done on the prevalence polypharmacy and probably inappropriate medications (PIM) and it associated risk factors. I can relate well with it but couldn’t do much to confirm the appropriateness of the statistical analysis employed. Hence, I have a few suggestions to make. |  |
| 1. There are a few grammatical errors that should be corrected. | We edited the entire manuscript and fixed any grammatical errors. We hope things are better now. |
| 2. The spelling of some of the medicines should be corrected. Eg. Benzodiazepine and Amiodarone. It would be appropriate if generic names were used for the specific examples of medicines names used in Table 3. | We revised the spelling of the medicines in table 3 and edited them. |
| 3. Briefly describe how the Beer’s criteria is used in such analysis instead of or in addition to the changes or update made to the 2015 version in your introduction.  4. Does it have any setbacks? | In the introduction, we added a paragraph that discusses the Beers criteria and how it was utilized and a small paragraph explaining its limitations. |
| 5. In all, how many PHC clinics did you conduct this study? | The study was carried out in three Palestinian West Bank districts of Nablus, Jenin, and Tukaram. We selected six PHC centers in each district, making a total of 18 clinics involved in the study.  We paraphrased this section in the methods section; we hope its clear now. |
| 6. Did all participants have creatinine level in their medical folders? How recent were they? | Yes, all of the participants had creatinine levels in their medical records, and we chose readings from the prior three months.  This was made clear in the methods section |
| 7. Nothing was said about the creatinine level and weight data collected in the patient’s medical records in the discussion. Or was it only to aid in the evaluation of medicines for Beer’s criteria? | Yes, the creatinine level and weight were used to compute creatinine clearance and GFR and aid in the evaluation of medicines for Beers criteria |
| 8. Apart from the Beer’s criteria, did you consider any current reference for medicines classification or status recommendations in terms of PIM? | The focus of this paper was solely on Beers criteria. However, this is an important consideration for future research. Thank you for pointing that |
| 9. Is any of the authors a practicing Pharmacist? | Yes, one of the authors (RA) is a practicing pharmacist in addition to being a lecturer at the Faculty of Medicine and Health Sciences' Pharmacy department, and two of the authors (ES and SN) are practicing family physicians working in primary health care centers. |
| 10. According to line 179, I think it wasn’t surprising that long-acting sulfonylureas was found to be the most frequent PIM. | The fact is that the drug is no longer recommended as a first-line treatment because to its side effects, particularly in this age group, as well as the advancement of diabetic treatments and safer alternatives, so we were somewhat surprised. However, we appreciate your point because sulfonylureas are still frequently used. |
| 11. Could you further clarify why psychiatric medications could not be assessed as PIM?. | One of the study's limitations was that psychiatry patients are treated in different PHC clinics with separate filing systems. In addition, not all patients were aware of their medications or willing to provide us with this information. This was acknowledged in the limitation section |
| **Reviewer 2 comments** | **Authors’ Response:** |
| Have the authors made all data underlying the findings in their manuscript fully available?  Reviewer #2: No | Thank you for bringing this to our attention; we have included the raw data in the supporting data files. |
| General: English language editing is needed. | The manuscript has undergone English language editing. |
| ***Methodology:***  Is a medication with two ingredients considered as one or two medications? | Medications with two different ingredients were treated as two medications; we intended to examine the PIM for each ingredient separately. We added this point in the methods section |
| ***Results:***  “The average number of total medications used per patient was six, ranging from zero to 15”. How were patients with zero medications included in the study? The inclusion criteria say: The study included all patients ≥ 65 years who had been taking medications for more than two weeks. | Thank you for drawing our attention to this point. There are no patients with a medication tally of zero, but the term was used in its statistical sense; zero to fifteen. We modified the sentence within the text. |
| **Table 2**: Is the number of patients using hypoglycemic agents identical to that who using antihypertensive agents? both are 226? | Thank you for bringing it up. No, they were not both identical. In table 2, we fixed this by including the number of hypoglycemic medications and their percentages. |
| **Table 3**: Add ‘first-generation’ antihistamines. | This has been added. |
| **Multiple regression**: I believe that the multiple regression model should not be limited to variables that showed significant bivariable association. This is because some of these variables can still affect the tendency of having the independent variable (PIM) especially if there is a significant difference in the distribution of this variable between people with and without PIM. | Thank you for notifying us to this. We agree with your statement, in that we carried out the logistic regression model using all factors shown to be significant on bivariable analysis as well as those considered necessary in the literature. Related changes are made to last part of the Data analysis paragraph in the methods section, as well as to table 4. |
| **Table 4**: Related to the above comment, the distribution of the independent variables (e.g., male/female) should be presented based on the dependent variable. i.e., in PIM group, how many males and females were among these 155 patients? The sum should be 100%. | The distribution of gender in relation to the PIM is presented in the second column of table 4. The sum of the percentages is not 100% because we utilized row percentages rather than column percentages; how many of the male patients have PIM compared to the females, and the total of the row percentage is 100%. |
| **Discussion:**  Compare the results of the most encountered PIMs (SU, alpha blockers and NSAIDs) with other studies.  The results should be compared to other studies done in Palestine even those used criteria other than Beers’ (e.g., https://www.dovepress.com/evaluating-inappropriate-medication-prescribing-among-elderly-patients-peer-reviewed-fulltext-article-CIA). | Thank you for bringing this study to our attention. We used it in the discussion and compared our results to theirs taking into consideration the difference in the setting and tools.. |
| “We believe that the findings of this study are generalizable to the elderly Palestinians attending PHC centers because of the relatively large sample size drawn from different districts of Palestine”  As the sampling technique was convenient rather than stratified randomized nationwide sampling, the above assumption is not valid. | We agree that the assumption was incorrect and have updated this section. |
| **Journal requirements** | **Authors’ Response:** |
| When submitting your revision, we need you to address these additional requirements. |  |
| 1. Please ensure that your manuscript meets PLOS ONE's style requirements, including those for file naming. The PLOS ONE style templates can be found at  <https://journals.plos.org/plosone/s/file?id=wjVg/PLOSOne_formatting_sample_main_body.pdf>  and  <https://journals.plos.org/plosone/s/file?id=ba62/PLOSOne_formatting_sample_title_authors_affiliations.pdf> | Done |
| 2. Thank you for stating the following financial disclosure:  "The author(s) received no financial support for this article's research, authorship, and/or publication."  At this time, please address the following queries:  a) Please clarify the sources of funding (financial or material support) for your study. List the grants or organizations that supported your study, including funding received from your institution.  b) State what role the funders took in the study. If the funders had no role in your study, please state: “The funders had no role in study design, data collection and analysis, decision to publish, or preparation of the manuscript.”  c) If any authors received a salary from any of your funders, please state which authors and which funders.  d) If you did not receive any funding for this study, please state: “The authors received no specific funding for this work.”  Please include your amended statements within your cover letter; we will change the online submission form on your behalf. | Done |
| 3. Thank you for stating the following in your Competing Interests section:  "The authors declare that they have no competing interests in this study."  Please complete your Competing Interests on the online submission form to state any Competing Interests. If you have no competing interests, please state ""The authors have declared that no competing interests exist."", as detailed online in our guide for authors at <http://journals.plos.org/plosone/s/submit-now>  This information should be included in your cover letter; we will change the online submission form on your behalf. | Done |
| 4. Please include captions for your Supporting Information files at the end of your manuscript, and update any in-text citations to match accordingly. Please see our Supporting Information guidelines for more information: <http://journals.plos.org/plosone/s/supporting-information>. | Done |
