# Supplementary material for: Potentially inappropriate medication uses and associated factors among elderly primary health care clinics attendees: A call to action
Source: PLoS One. 2023 Aug 24;18(8):e0290625. doi: 10.1371/journal.pone.0290625 (PMC10449131; doi:10.1371/journal.pone.0290625)
Supplement: Attachment — Submitted filename: Response to Reviewers.docx [file pone.0290625.s004.docx]

DATE: 10\08\2023

**Dear Dr. Muhammad Eid Akkawi**

**Academic Editor/ PLOS ONE**

RE: Manuscript entitled "Potentially inappropriate medication uses and associated factors among elderly primary health care clinics attendees: a call to action" ([PONE-D-23-10547] - [EMID:c0429b4b8a8af4b0]).

We appreciate your time and effort in evaluating our revised manuscript and responding to us.

We appreciate your comments and suggestions. We carefully read them and the Journal Requirements and responded to them point by point below.

We submitted one clean copy and a marked-up copy of our manuscript that highlights changes made to the original version. Our responses below are in the “Authors’ Response” column and shown in blue to distinguish them from the Reviewers’ comments.

We hope that we have adequately responded to your comments. We believe that this has strengthened our paper and look forward to hearing from you. Please let us know if you have any additional suggestions or if anything is unclear.

Many thanks for considering our revision for publication.

Sincerely,

Zaher Nazzal

| **Editor Comments:** | **Authors’ Response:** |
| --- | --- |
| **Abstract:**  probably inappropriate medication (PIM), potentially not probably.  aged 65 and up >> aged 65 and above.  Sulfonylureas being the most common>> were the most common  Polypharmacy [aOR= 4.1, 95%CI: 1.6 – 10.7], the number in brackets are related to hyperpolypharmacy (> 10 drugs) while for polypharmacy it is [aP-value =.003, aOR= 2.8, 95%CI: 1.4–5.4]. Please choose which one you want it to appear in your abstract.  one-third of the elderly attending >> of the elderly patients attending  Improving PHC clinics' physicians' awareness >> Improving physicians' awareness | We made edits to address the issues pointed out in your comments about the abstract. |
| **Introduction:**   - STOP to be corrected to STOPP throughout the manuscript. - “It was developed in 1991 by Mark Beers and has been updated every three years since 2011” not really every three years. Just change it to every few years. - benzodiazepine (14) >> benzodiazepines (14) - They are significantly affected by chronic diseases such as Diabetes, hypertension, congestive heart failure, atrial fibrillation, and coronary heart disease, most commonly with their medications furosemide, hydrochlorothiazide, and atorvastatin, levothyroxine, lisinopril, metoprolol, simvastatin, atenolol, amlodipine, and metformin>> Please paraphrase. - AGS>> should be written in full when it first appears. | Thank you for bringing this to our attention. We edited them all and paraphrased the section on chronic diseases among the elderly in Palestine. |
| **Methodology:**   - clinics in the Ministry >> clinics of the Ministry - In addition, most of the Palestinian are elderly (two-thirds) had governmental insurance rather than private or no insurance >> not clear. Please paraphrase. | We made the required edits and paraphrasing |
| **Results:**   - Most (90.5%) >> Most of them (90.5%) - The most common is hypertension >> The most common disease was hypertension - one-fourth have more than three >> one-fourth of them has more than three - The prevalence of PMI >> PIM | Done |
| - Table 2: to change the caption to “Distribution of the most commonly prescribed medications…” as the table does not show all medications used under each drug class. | Thank you for your suggestion. We changed the caption of table 2 as suggested. |
| **Discussion:**   - One of the reviewer’s comments was not addressed, which is “Compare the results of the most encountered PIMs (SU, alpha blockers and NSAIDs) with other studies”. The only added paragraph was the comparison with another Palestinian study. | Thank you for bringing this to our attention. In the discussion, we added a paragraph comparing the findings of the most commonly seen PIMs to those of other studies. |
| **Journal requirements** | **Authors’ Response:** |
| - Please review your reference list to ensure that it is complete and correct. If you have cited papers that have been retracted, please include the rationale for doing so in the manuscript text, or remove these references and replace them with relevant current references. Any changes to the reference list should be mentioned in the rebuttal letter that accompanies your revised manuscript. If you need to cite a retracted article, indicate the article’s retracted status in the References list and also include a citation and full reference for the retraction notice. | We appreciate your feedback. Yes, there have been some changes to the reference list. While paraphrasing the introduction and the discussion, it became clear that some resources needed to be added and others needed to be removed.  We revised our references and references list and ensured that all included references are relevant and all references included in the list were properly cited in the text. |
